# Structure and spectroscopy of CuH prepared *via* borohydride reduction

**DOI:** 10.1107/S2052520615015176

**Published:** 2015-11-07

**Authors:** Elliot L. Bennett, Thomas Wilson, Patrick J. Murphy, Keith Refson, Alex C. Hannon, Silvia Imberti, Samantha K. Callear, Gregory A. Chass, Stewart F. Parker

**Affiliations:** a School of Chemistry, Bangor University, Bangor, Gwynedd LL57 2UW, Wales; b ISIS Facility, STFC Rutherford Appleton Laboratory, Chilton, Didcot, Oxon OX11 0QX, England; c Department of Physics, Royal Holloway, University of London, Egham TW20 0EX, England; d School of Biological and Chemical Sciences, Queen Mary University of London, London E1 4NS, England

**Keywords:** copper(I) hydride, total scattering neutron diffraction, inelastic neutron scattering spectroscopy, X-ray powder diffraction

## Abstract

Copper(I) hydride (cuprous hydride, CuH) was the first binary metal hydride to be discovered (in 1844) and is singular in that it is synthesized in solution, at ambient temperature. There are several synthetic paths to CuH, one of which involves reduction of an aqueous solution of CuSO_4_·5H_2_O by borohydride ions. The product from this procedure has not been extensively characterized. Using a combination of diffraction methods (X-ray and neutron) and inelastic neutron scattering spectroscopy, we show that the CuH from the borohydride route has the same bulk structure as CuH produced by other routes. Our work shows that the product consists of a core of CuH with a shell of water and that this may be largely replaced by ethanol. This offers the possibility of modifying the properties of CuH produced by aqueous routes.

## Abstract 2

We show by a combination of diffraction and spectroscopic methods that CuH produced by borohydride reduction of a Cu^II^ salt consists of a core of CuH with the Wurtzite structure and a shell of water. We also demonstrate that the shell is exchangeable for ethanol.

## 1. Introduction   

In 1844 Adolphe Würtz reported to the Académie des Sciences (France) that the action of hypophosphorous acid on copper salts resulted in the formation of a copper hydride (Würtz, 1844 ▸). Copper(I) hydride (cuprous hydride, CuH) was the first binary metal hydride to be discovered and is singular in that it is synthesized in solution, at ambient temperature. It is also unusual in that it adopts the Würtzite structure (space group *P*6_3_ *mc*, No. 186; Goedkoop & Andersen, 1955 ▸); binary metal hydrides usually have the same crystal structure as the pure metal (Fukai, 1993 ▸), thus for CuH a face-centred cubic (f.c.c.) structure might be expected. An f.c.c. CuH is known but it is only stable above 12.5 GPa (Burtovyy & Tkacz, 2004 ▸). A second high-pressure phase, Cu_2_H, is formed at pressures above 18.6 GPa (Donnerer *et al.*, 2013 ▸). This compound exhibits an *anti*-CdI_2_-type structure (space group 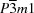 No. 164), where H atoms are proposed to occupy every second layer of octahedral interstitial sites.

Würtz prepared CuH by the reduction of acidic, aqueous copper sulfate with hypophosphorous acid. This remains the most often used route to CuH (Warf & Feitknecht, 1950 ▸; Fitzsimons *et al.*, 1995 ▸), although other routes have been developed including a sonochemical synthesis (Hasin & Wu, 2012 ▸), precipitation of the hydride from pyridine solutions of CuI and LiAlH_4_ (Dilts & Shriver, 1968 ▸) and reduction of aqueous Cu^2+^ by borohydride ions (Dasgupta & Mahanti, 2010 ▸), see equation (1).
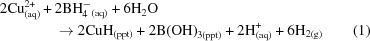
While the products from the Würtz and the pyridine methods have been extensively investigated (Korzhavyi *et al.*, 2012 ▸; Soroka *et al.*, 2013 ▸; Auer & Kohlmann, 2014 ▸; Bennett *et al.*, 2015 ▸), that produced *via* equation (1) has been much less studied. This synthetic method results in an insoluble form of CuH, (‘CuH/BH_4_’), that is coffee-coloured rather than the rust-red obtained from the Würtz synthesis. This form also appears to be more stable than the others because it is stated that it survives in boiling water, whereas the products from the other routes decompose therein. The CuH/BH_4_ product is reported to show an IR band at 521 cm^−1^, which was assigned to a Cu⋯H⋯Cu⋯H bridge structure (Dasgupta & Mahanti, 2010 ▸). However, CuO is a common product of the aerial decomposition of CuH and it exhibits a strong IR band at 537 cm^−1^ (Kliche & Popovic, 1990 ▸) and this is a more reasonable assignment. CuH/BH_4_ was recently studied by X-ray and neutron powder diffraction (Auer & Kohlmann, 2014 ▸). The aims of this paper are to use a combination of structural and spectroscopic methods to provide further insight into the nature of CuH formed *via* the borohydride route.

## 2. Experimental   

### 2.1. Synthesis   

CuH/BH_4_ was prepared by a variation of the literature synthesis (Dasgupta & Mahanti, 2010 ▸) based on equation (1). In a two-necked 1 L round bottom flask, CuSO_4_·5H_2_O (40 g, 160.2 mmol, Aldrich, 99.995%) was fully dissolved in a minimum of 2 *M* H_2_SO_4_. The aqueous mixture was degassed with argon for 2 h and warmed slowly to 303 K upon which a solution of NaBH_4_ (6.06 g, 160.2 mmol, Aldrich, 99%) was added slowly. The vigorous exothermic reaction instantaneously resulted in coffee-coloured particles of CuH and flecks of copper metal. Under an inert argon atmosphere the reaction vessel was then sealed with rubber septa and stirred at 303 K for 2 h. The mixture was filtered through a sintered glass filter of medium porosity (G02) under a flowing argon blanket. The filtrate was blue due to the presence of unreacted aqueous Cu^II^. The CuH cake was washed with distilled, degassed room temperature water to remove any remaining soluble starting materials, followed by distilled water at 333 K to remove free boric acid crystals that form upon cooling. The product was then washed consecutively with degassed ethanol and degassed diethyl ether and dried by argon aspiration. [The ethanol was used to remove any excess water and the ether to remove any residual ethanol left and to aid the drying process (argon-aspiration).] After being filtered, washed and dried, the product and the sintered glass filter was transferred into a sealed argon-filled glove bag. It was then weighed (7.8 g) and split into two batches. One batch was loaded into the sample holders for neutron characterization (TOSCA and SANDALS). A small amount (< 0.3 g) was taken and washed with ether (10 ml) in a small round bottom flask, and then filtered under argon to remove residual water then dried under argon aspiration to obtain a free-flowing dry powder which could be loaded into a 1 mm quartz capillary for XRD analysis.

The second batch of the CuH product (≃ 3.5 g) was stirred for ∼ 12 h in D_2_O under an argon atmosphere, then filtered, dried *via* vacuum filtration and, as before, loaded into sample holders for neutron studies.

**Caution:** *Note that the exclusion of air is essential as the dried product may decompose explosively.*

### 2.2. Neutron diffraction   

The CuH sample was loaded into flat-plate 40 mm diameter cells of 1 or 2 mm thickness made of the null scattering alloy Ti(52.5%)Zr(47.5%). Time-of-flight neutron diffraction measurements were performed using the diffractometer SANDALS (http://www.isis.stfc.ac.uk/instruments/sandals/) at ISIS over the range 0.1 ≤ *Q* ≤ 50 Å^−1^ at room temperature. A full set of experimental corrections and an absolute normalization were made using the standard *Gudrun* software (ISIS, 2015 ▸).

### 2.3. X-ray diffraction   

X-ray diffraction (XRD) measurements were made at room temperature using a Panalytical X’Pert Pro multi-purpose diffractometer in capillary mode, with a silver anode source (wavelength 0.560885 Å).

### 2.4. Inelastic neutron scattering (INS) spectroscopy   

The CuH samples were either loaded (in a glovebag) into sealed aluminium cans or the same samples that were used for the SANDALS measurements in their TiZr cans were loaded into a closed cycle cryostat and cooled to ∼ 20 K. The INS spectra were then recorded with the high resolution (∼ 1.25% of the energy transfer) spectrometer TOSCA (Parker *et al.*, 2014 ▸) at ISIS for 8–12 h. (The spectra were recorded at low temperature in order to minimize the Debye–Waller factor; Parker *et al.*, 2011 ▸.)

## 3. Results and discussion   

The synthesis of CuH *via* the borohydride route has not been extensively studied. Equation (1) requires a 1:1 stoichiometry of CuSO_4_ to NaBH_4_, but it was clear from the deep blue colour obtained of the filtrate that the limiting factor in this reaction is the NaBH_4_. It is also worth noting that increasing the quantity of the reactants (*i.e.* 160.2 mmol → 400.5 mmol) in the hope of increasing the mass yield gave rise to filtering and aspiration problems.

**Figure 1:**
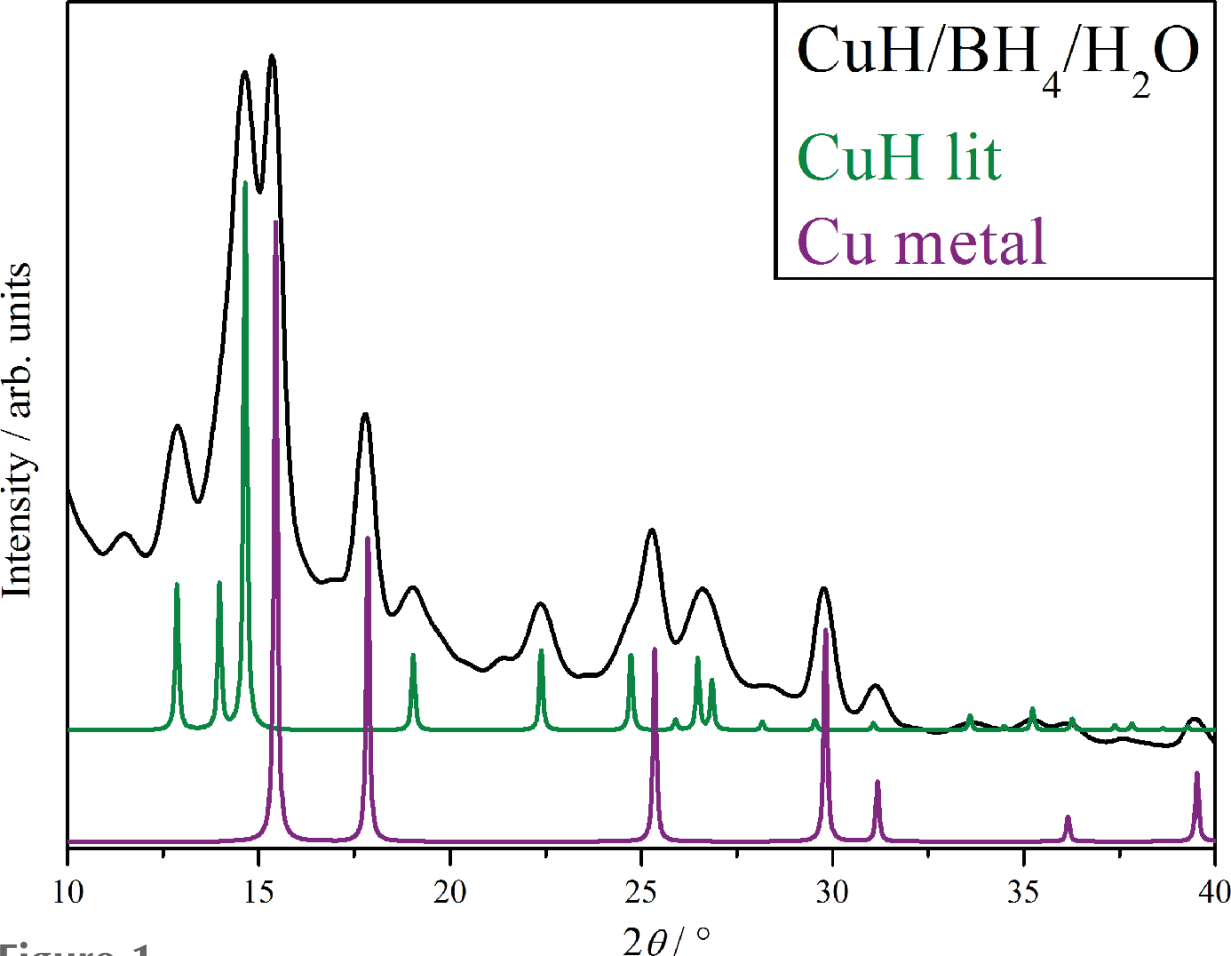
Comparison of the XRD pattern of CuH as prepared by the borohydride method (black) and simulated patterns of CuH (olive green) and Cu metal (purple). The weak peaks at 11.5 and 21.3° are assigned to small quantities of Cu_2_O and/or CuO.

The vigorous, effervescent and exothermic reaction that occurs upon addition of the NaBH_4_ generates sufficient localized heating to allow small amounts of decomposition to copper metal. Minute, but visible, amounts of copper metal can been seen in the reaction mixture and in the dried product, as well as visible crystals of B(OH)_3_. The literature (Dasgupta & Mahanti, 2010 ▸) states that full removal of this free boric acid can be achieved by boiling the crude CuH product (after filtration) in boiling water for 1 h. Addition of H_2_O (deionized and degassed) at 343–348 K instantly resulted in effervescence and CuH decomposition, thus an hour in boiling water would result in complete decomposition, with the most likely product being CuO. The coffee colour of the product can be ascribed to it being a mixture of CuH and Cu metal. XRD of the washed product, Fig. 1 ▸, confirms the presence of CuH and a substantial quantity of Cu metal.

**Figure 2:**
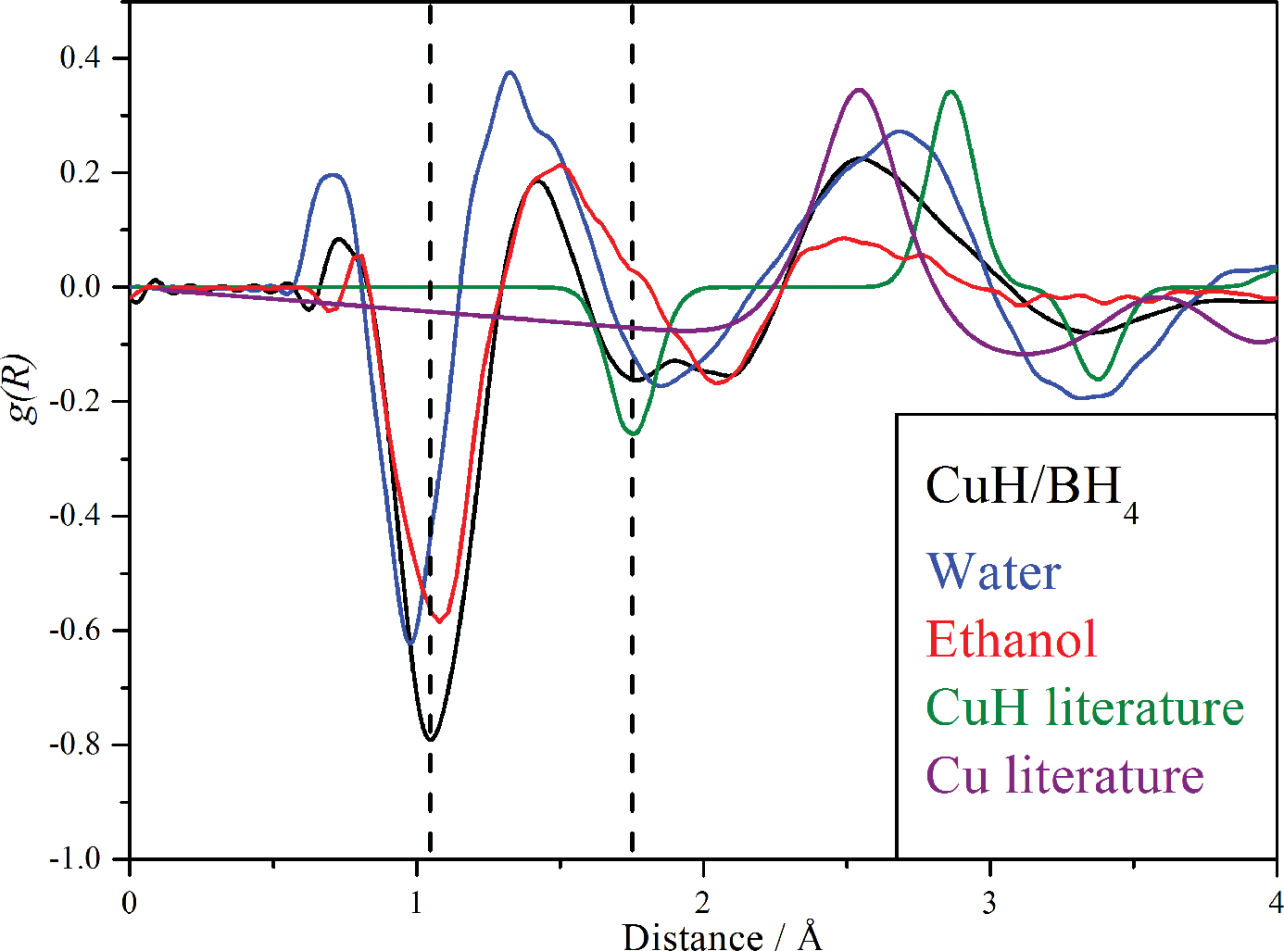
Comparison of radial distribution functions from neutron diffraction data for CuH prepared by the borohydride route (black), liquid water (blue), liquid ethanol (red) and those calculated from the literature structure for CuH (olive green) and Cu metal (purple). The dashed vertical lines are at 1.05 and 1.76 Å.

Neutron total scattering using the SANDALS diffractometer allows direct observation of the characteristic Cu—H distance of 1.78 Å *via* generation of the radial distribution function *g*(*R*). As shown in Fig. 2 ▸, there is clearly a negative-going peak at 1.76 Å confirming the presence of CuH. There is also a substantial peak at ∼ 1.05 Å due to an *X*—H link. We will discuss this later in the paper.

**Figure 3:**
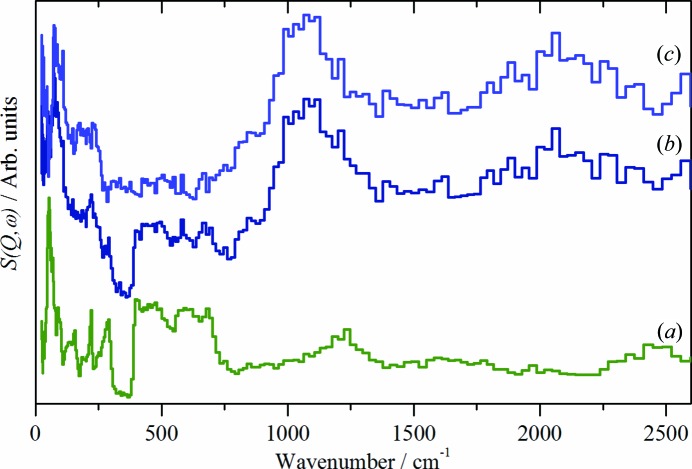
INS spectra recorded on TOSCA at 20 K of: (*a*) D_2_O, (*b*) CuH/BH_4_ after stirring in D_2_O and (*c*) the scaled difference spectrum. (*a*) is plotted on a different ordinate scale to that of (*b*) and (*c*).

INS spectroscopy is a complementary form of vibrational spectroscopy (Mitchell *et al.*, 2005 ▸) The scattering intensity depends on the incoherent inelastic scattering cross section and the amplitude of vibration. For ^1^H both of these are large, consequently the scattered intensity is dominated by hydrogenous motion. Neutrons are highly penetrating, so the spectra are representative of the bulk rather than just the surface. The use of INS spectroscopy allows the Cu—H vibration to be observed. Fig. 3 ▸ shows a comparison of the CuH product after isolation and subsequent stirring with D_2_O, a reference spectrum of D_2_O ice *I*_h_ and the difference spectrum. The difference spectrum shows a strong peak at 1070 cm^−1^ and a weaker peak at 2080 cm^−1^. These are assigned as the 0 → 1 and 0 → 2 transitions of CuH (Bennett *et al.*, 2015 ▸). Comparison of the spectra of solid D_2_O with the exchanged product clearly shows the presence of D_2_O ice *I*_h_ in the CuH product, but also that the CuH has not exchanged. This strongly supports a core-shell model comprised of a CuH core and a water shell. While there is a substantial amount of copper metal present, its contribution to the spectrum is small because the total scattering cross section of Cu is 8.0 barns *versus* 90.0 barns for CuH. Further, the Cu metal phonons all occur below 400 cm^−1^ (Nicklow *et al.*, 1967 ▸) so do not contribute to the spectrum in the region of the Cu—H stretching modes at ∼ 1000 cm^−1^.

**Figure 4:**
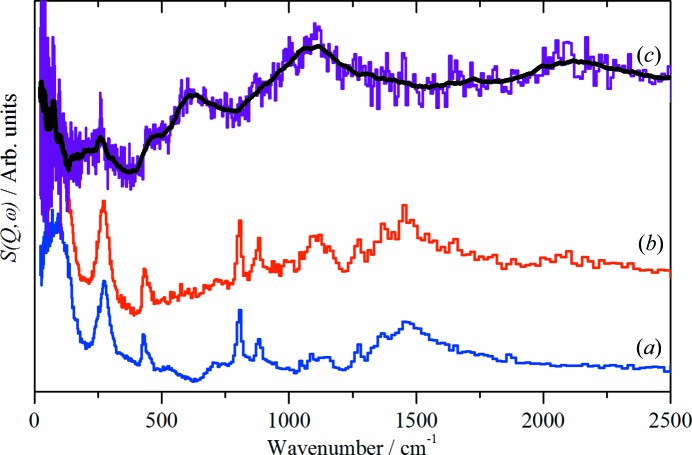
INS spectra recorded on TOSCA at 20 K of: (*a*) ethanol, (*b*) CuH prepared by the borohydride route and (*c*) the scaled difference spectrum. (*a*) and (*b*) are plotted on different ordinate scales, (*c*) is 10 times expanded relative to (*b*). The black line in (*c*) is the smoothed spectrum as a guide to the eye.

**Figure 5:**
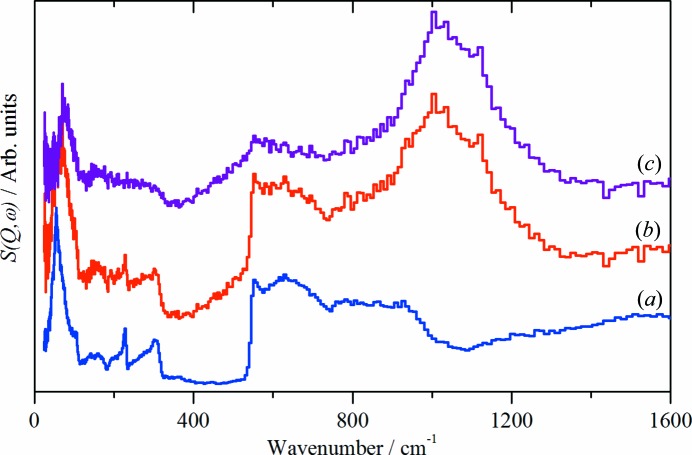
INS spectra recorded on TOSCA at 20 K of: (*a*) crystalline ice *I*_h_, (*b*) CuH prepared by the Würtz route and (*c*) the scaled difference spectrum.

Fig. 4 ▸ shows a comparison of the CuH product as initially isolated: *i.e.* after washing with ethanol, a reference spectrum of ethanol and the difference spectrum. In agreement with the spectra in Fig. 3 ▸(*c*), the difference spectrum shows a strong peak at 1070 cm^−1^ and a weaker peak at 2080 cm^−1^. These are assigned as the 0 → 1 and 0 → 2 transitions of CuH. The unexpected feature is that the isolated product shows no evidence of water, only ethanol is apparent. The difference spectrum, Fig. 4 ▸(*c*), does show a weak feature at 625 cm^−1^ that is assigned to the librational modes of a small amount of residual, strongly disordered water. Since the procedure used for isolation of CuH prepared by the Würtz method (Bennett *et al.*, 2015 ▸) was the same as that employed here, it follows that there should be strongly bound water present in samples prepared by this route. Fig. 5 ▸ shows that this is the case: after subtraction of ice *I*_h_ (Fig. 5 ▸ *a*), from the as-prepared sample (Fig. 5 ▸ *b*), the difference spectrum (Fig. 5 ▸ *c*) shows the translational and librational modes of disordered water. Assuming a particle size of 10 nm (Fitzsimons *et al.*, 1995 ▸), we estimate that the amount of disordered water present corresponds to a few monolayers.

Fig. 4 ▸ suggests that the water shell enduring from the synthesis has been largely exchanged for ethanol by the washing process. This is the first example where it has been possible to exchange the outer shell of CuH prepared by an aqueous route for anything other than D_2_O. This proposal is supported by the neutron diffraction data shown in Fig. 2 ▸; the apparent 0.1 Å difference between the O—H distance in liquid water and the water in the shell of CuH is very difficult to rationalize, but it is readily understandable if the peak in the CuH sample is due to ethanol, where the peak will be dominated by the C—H distance of ∼ 1.1 Å, rather than the O—H distance of ∼ 1.0 Å. In contrast to water, where the shell freezes to crystalline ice *I*_h_, the ethanol shell remains amorphous, as shown by the broad methyl torsion at 275 cm^−1^ in both the reference spectrum and that of CuH.

**Figure 6:**
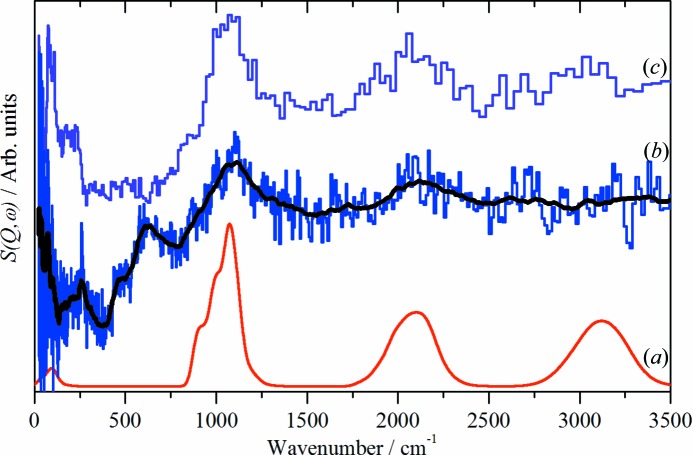
Comparison of INS spectra of: (*a*) CuH calculated using *CASTEP* and generated using a relaxed instrumental resolution function, (*b*) the initially isolated CuH prepared by the borohydride route after subtraction of ethanol and (*c*) the product in (*b*) after exchange with D_2_O and subtraction of D_2_O.

We have shown elsewhere (Bennett *et al.*, 2015 ▸) that the difference between the products from the Würtz method and from the non-aqueous method is the nature of the surface termination of the CuH particles: chemically bound hydroxyls and coordinated pyridine, respectively. The INS spectrometer used in this work does not allow observation of the hydroxyl O—H stretch, but our previous computational studies show that the Cu—O—H bond-angle deformation modes occur in the 500–800 cm^−1^, where there is a pronounced shoulder that is not predicted for CuH. Inspection of Figs. 6 ▸(*b*) and (*c*) shows that CuH from the borohydride route also exhibits such a shoulder, consistent with the model that CuH produced from aqueous solution is hydroxyl terminated. The presence of the disordered water indicates that CuH particles from aqueous routes consist of a crystalline core of non-stoichiometric CuH terminated by hydroxyls with a few layers of disordered water and an outer layer of unperturbed water that freezes to crystalline ice *I*_h_.

We have previously calculated (Bennett *et al.*, 2015 ▸) the INS spectrum of CuH by periodic density functional theory using the *CASTEP* code (Clark *et al.*, 2005 ▸). The identification of the difference spectra as being CuH is confirmed by comparison to the calculated INS spectrum shown in Fig. 6 ▸. It is also clear that the CuH core is unchanged by modifying the shell through successive replacement of water with ethanol and D_2_O.

## 4. Conclusions   

The combination of diffraction and vibrational spectroscopic studies clearly demonstrate that the CuH product resulting from synthesis by the borohydride route [equation (1)] is the same as that generated by the traditional synthesis based on Würtz’s 1844 method. This is in agreement with the most recent powder diffraction study (Auer & Kohlmann, 2014 ▸). In particular, the reported differences (Dasgupta & Mahanti, 2010 ▸) between the products from the Würtz and the borohydride routes; the colour and the relative stability are artefacts and are incorrect. The colour is due to a significant admixture of copper metal and our experience was that the borohydride product was no more stable than that produced by other routes, *i.e.* it decomposed within a few days at room temperature.

Our discovery that the water shell may be partially replaced by ethanol is significant because (other than with D_2_O) this is the first time that this has been achieved. The properties of CuH are strongly dependent on the surface chemistry and this opens possibilities for modifying the properties of CuH produced by aqueous routes.
